# Decolonizing end-of-life care: lessons and opportunities

**DOI:** 10.3332/ecancer.2022.ed121

**Published:** 2022-04-28

**Authors:** Christian Ntizimira, Mbonyinkebe S Deo, Mary Dunne, Eric Krakauer

**Affiliations:** 1 Executive Director, African Center for Research on End-of-Life Care, KK 394, KK 43 Av, Kicukiro Niboye, PO Box 00000, Kigali, Rwanda; 2 Fellow, Rwanda Academy of Sciences, Ituze House, 3rd Floor, KN Rd 6 Remera, PO Box 00000, Kigali, Rwanda; 3 Fellow, Distinguished Career Institute, Stanford University, 450 Serra Mall, Stanford, CA 94305, USA; 4 Assoc Professor of Medicine and of Global Health & Social Medicine, Harvard Medical School, 641 Huntington Ave, Boston, MA 02115, USA

**Keywords:** palliative care, end-of-life, culture, Africa, colonialism

## Abstract

While palliative care should be universally accessible [1], the specific types and severity of illness and suffering vary by geopolitical situation, socioeconomic condition, and culture [2]. The meaning of suffering and death vary similarly [3]. As such, palliative care should consider local culture when considering the needs of individual patients and families. While pain and symptom control have universal value, optimal application may vary greatly depending on context.

This work was presented at the Palliative Care, Culture and the Clinic symposium in January 2021 and the accompanying Special Issue from the event can be found here: https://ecancer.org/en/journal/special-issue/32-biomedicine-and-the-soul-in-relation-to-global-palliative-care.

## Background

The modern hospice movement and the specialty of palliative medicine evolved in developed countries and have been applied to developing countries using the Western medical model [4] But how do people die who are not fully assimilated to Western culture? Consider non-acculturated people throughout Africa and Asia, for example, and indigenous peoples of persistently colonized countries. Are the needs of the dying in Rwanda, Rajasthan, or on the Rose Bud Reservation the same as White middle-class Parisians or Pennsylvanians? Do health and illness, suffering and death, life and after-life, have the same meaning everywhere? These questions are rhetorical, as the clear answer to all is “no.” For some, wakefulness in the final moments is crucial even if pain must be endured, while others wish to be pain-free even at the cost of alertness. Some want nothing more than to be at home surrounded by family at the end, others want to fight for longer life in the ICU.

While the World Health Organization (WHO) asserts that palliative care should be universally accessible [1], it has recently recognized that the specific types, severity and meanings of suffering vary by geopolitical situation, socioeconomic conditions and culture [2,3]. Thus, research is needed to understand local attitudes to infirmity, suffering, dying, and death. [5] (WHO 2018).

Assuming that the palliative care practiced in Paris and Pennsylvania is the only medically and ethically correct palliative care model represents an intellectual knowledge that again places the West in the role of bringing enlightenment to those living in the dark. Before the European colonizers brought “enlightenment” (and unspeakable destruction) to the rest of the world, there were cultural values and practices to make meaning and minimize the suffering of dying and death, many of which persist today [4]. We propose that the field of palliative care, with its emphasis on listening, should endeavor to listen carefully to the voices of traditions that may provide meaning and comfort not achievable with modern technological means alone [6] (Taylor 2020).

Across the African continent, there is a need to rethink palliative and end of life care, to take account of traditional values and practices, and to find ways to integrate them with modern therapies. We may need also to rethink outside of the “colonial library” [7] to find local solutions to challenges identified in the community that would bring a constructive balance between culture and modernity. The Global Institute of Psychosocial, Palliative and End-of-Life Care (GIPPEC) at Princess Margaret Cancer Centre at the University of Toronto and the Institute of Cancer Policy, King’s College London have established a global long-term project to incorporate local social-cultural aspects into oncology and palliative care At a January, 2021, conference at the Dalla Lana School of Public Health of the University of Toronto, the African Center for Research on End-of-Life Care (ACREOL) shared its perspective on decolonizing palliative care by emphasizing an inclusive model of care, one which integrates both traditional and modern practices.

## “*Ubuntu*” as social justice and equity

Ernest Hemingway, the American novelist and Nobel Prize winner wrote: *“Every man’s life ends the same way. It is only the details of how he lived and how he died that distinguish one man from another.”* The details matter. The socio-cultural context matters. The perception of life, death and dying is like a fingerprint, unique to every human being and to every culture, community and society.

*Ubuntu* is the African idea of who we are. It is summarized in the expression: *Umuntu Ngumuntu Ngabantu,* that is, we are who we are through others.[8] To be dependent on others does not denote a loss of autonomy. Simply put, it reflects an understanding that a community is like a solid ancient arch, in which the stability of the whole depends on each stone. Ubuntu is an indicator of social justice and equity as well as a philosophy.

## Traditional end-of-life care existed before modern medicine

Modern palliative care, like Western medicine in general, objectifies the disease, the symptoms and the patients. This objectification risks discounting potentially beneficial non-Western practices and values, such as *Ubuntu*, the value of being through others [9]. The purpose of decolonization is not to pit developing and developed countries against each other, but to encourage a transcultural synergy that takes into account essential socio-cultural and traditional elements, existing practices, and local context.

It is true that access to pain and symptom control is increasing far too slowly in Africa, but a good end of life does not depend solely on medical interventions. It is important to research and recognize traditional ways of making the end of life comfortable and meaningful.

A consequence of the Covid-19 pandemic on countries with limited resources has been a decrease in funding for palliative care projects. Although many funded services shut down, palliative care continued because in Africa, in the spirit of *Ubuntu*, care is driven by commitment and community responsibility to others. As we say in Rwanda: “*When you are well, you belong to yourself but when you are sick, you belong to your family[community]”*

## International standards in palliative care: a trap

International standards in palliative care can themselves become a source of social injustice if they do not incorporate the values and needs of patients and families in all settings. Palliative care metrics are ethnocentric if they do not measure what benefits patients most in each context. Highly meaningful and comforting aspects of end-of-life care may not be recognized by Western measures, just as Western palliative care might seem lacking by some African standards. Perhaps Africa, Asia, and indigenous peoples have rich socio-cultural resources that could benefit the global community. We need to know.

## Conclusions

For equitable and contextually relevant palliative care, culture matters. Ignorance of the ideologies and values of patients and families creates misunderstandings and barriers to optimal care. Anthropological approaches suggest that understanding health in cultural context is key to the practice of socially conscious medicine through a whole-person/whole-people approach [10]. Palliative care should ensure that interventions are contextually relevant to meet the needs of each patient. In the name of equity, palliative care must be decolonized.

## Conflicts of interest

The authors declare no conflicts of interest with respect to the research, authorship and/or publication of this article.

## Funding declaration

The author(s) received no financial support for the research, authorship, and/or publication of this article.
